# A Catalog of Patent Applications Published in 2024 Related to Ophthalmology and Vision Science

**DOI:** 10.1167/tvst.14.5.16

**Published:** 2025-05-13

**Authors:** Guillaume Stern, Benjamin Stern, Jonathan J. Mallett, Roy S. Chuck

**Affiliations:** 1 Reinhold Cohn Group, Tel-Aviv, Israel; 2 Division of Ophthalmology, Hadassah Medical Center, Faculty of Medicine, Hebrew University of Jerusalem, Israel; 3 Anterior Segment and Refractive Surgery Department, Rothschild Foundation Hospital, Paris, France; 4 Senior Director of Journals, ARVO; 5 Albert Einstein College of Medicine, Bronx, NY, USA; 6 Editor-in-Chief e-mail: gustern@rcip.co.il

Over the last 2 years, we published in *Translational Vision Science and Technology* two articles^1^^,^^2^ providing detailed catalogs of international patent applications published in 2022 and 2023 by the World International Patent Office (WIPO) that were identified as being related to the field of vision science. We continue the series here by providing a listing of relevant applications published in 2024. For a more detailed description of the motivation and methodology, please see the original article in the series.^1^

As in the previous articles, the 2024 catalog is provided as a Supplementary Excel file, which allows it to be downloaded and readily searched, sorted, or filtered within Excel. Fields include titles, abstracts, and thumbnail images for each of the identified 1393 international patents, as well as International Patent Classification (IPC) class and group descriptions and patent application and publication dates.

We present below updates based on new data for the two figures we published in the previous articles. Figure 1 shows the number of ophthalmology-related applications by the top 20 applicants, and Figure 2 shows how those applications are distributed across technical fields for those top applicants.

**Figure 1.**
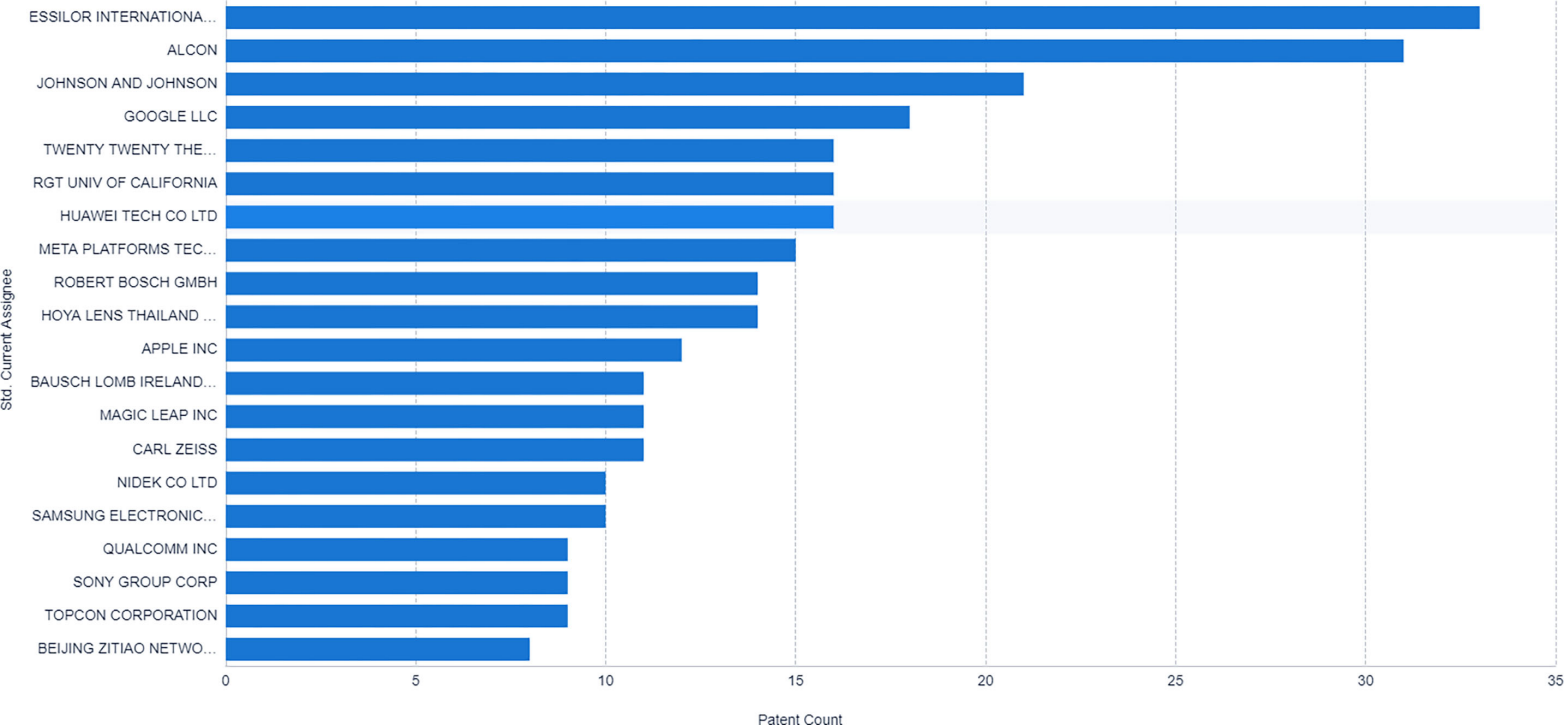
Number of vision science–related patent applications in 2024 by applicant for the top 20 applicants.

**Figure 2.**
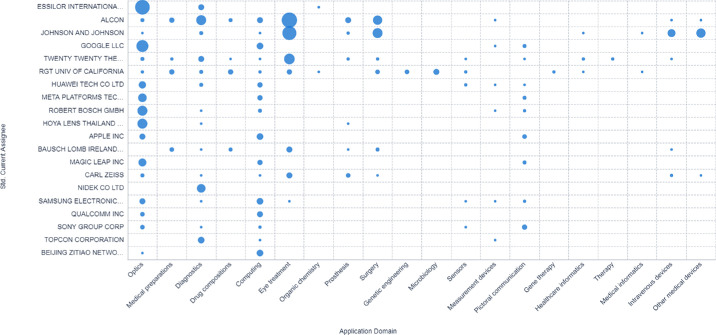
Distribution of 2024 patent applications across technical fields.

## Supplementary Material

Supplement 1
